# Low potassium disrupt intestinal barrier and result in bacterial translocation

**DOI:** 10.1186/s12967-022-03499-0

**Published:** 2022-07-06

**Authors:** Haishan Wu, Rong Huang, Jinjin Fan, Ning Luo, Xiao Yang

**Affiliations:** 1 Department of Nephrology, The First Affiliated Hospital, Sun Yat-Sen University, Guangzhou, 510080 China; 2 Key Laboratory of Nephrology, National Health Commission and Guangdong Province, Guangzhou, People’s Republic of China

**Keywords:** Low potassium, Gut permeability, Intestinal barrier, Dysbacteriosis, Tight junction

## Abstract

**Background:**

Bacterial translocation was observed in critical illness and patients with chronic diseases such as liver cirrhosis and chronic kidney disease (CKD). Hypokalemia is a common complication in these diseases. Whether low potassium diet may increase intestinal permeability and result in bacterial translocation lack of evidence. The present study was aimed to investigate the potential effects of LK on intestinal permeability.

**Methods:**

Grade 8-week-old male Bal B/C mice were randomly placed either on a normal potassium (NK) mouse chow or a low potassium (LK) diet for 28 days. Intestinal permeability and expression of tight junction proteins were compared between the two groups.

**Results:**

Compared with the NK group, the mice in LK group had significantly lower serum potassium level, increased levels of plasmas endotoxin and plasma d-lactate. The bacterial translocation was higher and in occurred mainly in mesenteric lymph nodes (MLN), liver and spleen. The pathologic change of small intestine was obvious with thinner villus lamina propria, shorter crypt depth and thinner intestinal wall. Slight increases in the expression of proteins and mRNA levels of both claudin-1 and claudin-2 were observed in LK group.

**Conclusions:**

Low potassium diet could increase intestinal permeability and thereby lead to bacterial translocation, which was suspected to result from impaired intestinal epithelial barrier and biological barrier.

**Supplementary Information:**

The online version contains supplementary material available at 10.1186/s12967-022-03499-0.

## Introduction

The bacterial environment of the intestinal tract has been investigated for its role in health maintenance and relationship to various disease states [1, 2]. Deterioration of intestinal barrier integrity affects gut permeability, resulting in bacterial translocation (BT). Enteropathogenic bacteria are the source of BT following dysbiosis and compromised barrier function [3], leading to endogenous infection and initiation of uncontrolled systemic inflammatory response syndrome that progress into multiple organ dysfunction syndrome [4]. BT is observed in critical illness [5] and patients with chronic diseases such as liver cirrhosis [6] and chronic kidney disease [7]. Spontaneous bacterial peritonitis is a common and life-threatening complication in patients with cirrhosis [8], whose occurrence and development are related to BT, especially of intestinal Gram-negative enterobacteria [9]. There is evidence that intestinal permeability increases in patients with uremia, and transcellular and intracellular protein constituents of colonic epithelial tight junctions (TJs) in rats induced by 5/6 nephrectomy are depleted [10]. Due to the risk of hyperkalemia in CKD, limiting potassium intake is often recommended. Hypokalemia, defined as potassium concentration < 3.5 mmol/l, affects 1–3% of the general population and patients with chronic kidney disease, and is more common (5–22%) among persons undergoing peritoneal dialysis (PD) [11], consequence of insufficient potassium intake and use of glucose dialysate. Peritonitis is a common and serious complication of PD [12] and patients with hypokalemia have a higher risk of PD-related peritonitis [13], but potential mechanisms for this phenomenon are not clear.

Permeability is one component of intestinal barrier function [14]. TJ proteins and their interactions tightly regulate paracellular permeability by facilitating movement of water and solutes between cells, while blocking the translocation of larger antigens [15]. TJ structure and stability can be regulated by several intrinsic and extrinsic factors including cytokines and growth factors, cellular stress, pathogens, probiotics, and dietary peptides [16]. Na–K-ATPase has been shown to play a fundamental role in the formation and maintenance of epithelial TJs [17]. Potassium is an essential electrolyte that is involved in various cellular homeostatic functions, including regulation and maintenance of cell volume, enzyme activity and DNA/protein synthesis [18, 19]. Decreased potassium concentration in monocyte macrophages is a factor in the activation of Nod-like receptor protein-3 inflammasomes. Classical activators of inflammasomes such as bacterial toxins and mitogen α-toxins are activators of potassium channels, which result in potassium outflow and depletion, inflammasome activation, and defense response. Hence, low intracellular potassium may be the least common trigger of inflammasome activation [20, 21].

It is still unknown whether low potassium (LK) increases intestinal permeability and then leads to BT. Hence, the present study investigated the potential effects of LK on intestinal permeability.

## Materials and methods

### Animals

Healthy 8-week-old male Bal B/C mice were obtained from Guangdong Medical Experimental Animal Center (Guangzhou, China). Mice were housed in an environment of constant temperature (25 °C) and humidity in a 12–12 h light–dark cycle with free access to a specific pathogen-free laboratory diet and distilled water during all the experiments. After 1 week of acclimatization, weight-matched mice were randomly placed on a normal potassium (NK) mouse chow or a LK diet (Diet Research, New Brunswick, USA). All animals were killed on days 7, 14, 21 and 28 after treatment. Mice were intraperitoneally anesthetized with chloral hydrate and blood specimens were obtained by eye enucleation.

### Influence of LK on intestinal movement in mice

Mice were administered activated carbon by gavage and the propulsion distance of activated carbon in the small intestine was detected after 30 min.

### Plasma and tissue collection

Blood was obtained by eye enucleation. Luminal contents were removed from the ileum and cecum and flash frozen in liquid nitrogen. Samples of mesenteric lymph nodes (MLN), liver, spleen, kidney and intestine were rapidly harvested and processed. One half of the intestine was fixed in 4% buffered paraformaldehyde (pH 7.4) at 4 °C overnight and embedded in paraffin for morphological staining and immunohistochemical analysis. Other sections were frozen in liquid nitrogen immediately for subsequent evaluation.

### Measurement of intestinal permeability in mice

Microbiota DNA were extracted from ileal and cecal luminal contents using the QiAamp DNA Stool Mini Kit (Qiagen, Hilden, Germany). Amplification was performed on the V4 region of the 16S rRNA genes via polymerase chain reaction (PCR) using the FastStart Universal SYBR Green Master (ROX) Kit (Roche, Basel, Switzerland) with an ABI Prism 7900 H T Sequence Detection System (Life Technology, Carlsbad, CA, USA). The primer sequences for PCR analysis are shown in Additional file 1: Table S1.

### Barrier function assessment

The paracellular and transcellular pathways were measured as the flux of 4 kDa fluorescein isothiocyanate-dextran (FD-4; Sigma-Aldrich, St. Louis, MO, USA). Mice were gavaged with FD-4 (1 ml/100 g) and blood samples were collected after 30 min. Concentration of FD-4 was measured via fluorescence at excitation 485 nm and emission 528 nm.

### Plasma lipopolysaccharide-binding protein

Lipopolysaccharide-binding protein levels were detected in plasma samples by ELISA (Cusabio Life Sciences, College Park, MD, USA).

### Cell cultures

Caco-2 cells were purchased from the American Type Culture Collection and maintained at 37 °C in a culture medium composed of Dulbecco’s Modified Eagle’s medium with 4.5 mg/ml glucose, 50 U/ml penicillin, 50 U/ml streptomycin, 4 mM glutamine, 25 mM HEPES, and 10% fetal bovine serum. The cells were kept at 37 °C in a 5% CO_2_ environment. Culture medium was changed every 2 days. Caco-2 cells were subcultured after partial digestion with 0.25% trypsin and 0.9 mM EDTA in Ca^2+^- and Mg^2+^-free phosphate-buffered saline (PBS).

### Antibodies

The following antibodies were used: rabbit claudin-1 polyclonal antibody (Abcam, Cambridge, MA, USA), rabbit claudin-2 polyclonal antibody (Abcam), rabbit occludin polyclonal antibody (Abcam), mouse β-actin monoclonal antibody (Cell Signaling Technology, Beverly, MA, USA). Horseradish peroxidase (HRP)-conjugated anti-mouse IgG, HRP-conjugated anti-rabbit IgG and Alexa-Fluor-488-conjugated anti-rabbit IgG were from Cell Signaling Technology.

### Serum biochemistry

Serum was isolated from blood by centrifugation (1000 rpm at 4 °C for 10 min). Serum potassium were measured by a Hitachi 7180 biochemistry autoanalyzer (Yokohama, Japan).

### Intestinal histopathology

Paraffin-embedded intestines were cut into 2-μm sections and serial 6-μm sections. The 2-μm sections were stained with hematoxylin–eosin (HE). Immunofluorescence analysis was performed on the serial 6-μm sections. The HE-stained tissue sections were used to assess the morphological changes in the intestinal wall.

### Measurement of transepithelial electrical resistance and paracellular permeability in vitro

Caco-2 cells were seeded and grown on collagen-coated polycarbonate membrane Transwell inserts with 0.4-μm pore size (Corning, NY, USA). Cells were allowed to grow until a transepithelial electrical resistance (TER) of > 350 Ω cm^2^ developed, usually after 19–21 days, and monolayers were exposed to LK medium. An epithelial voltohmeter (Millipore, Bedford, MA, USA) was used for measurements of the TER of the filter-grown Caco-2 intestinal monolayers. To obtain the TER values, the background resistance value of a blank filter without cells was subtracted from the measured values and then the values were normalized to the area of the filter. Confluent monolayers of Caco-2 cells with TER values ≥ 350 Ω cm^2^ were chosen for these studies. Cells in culture medium, treated with control or LK conditions, were assessed for paracellular diffusion of fluorescent *Escherichia coli* (K-12 strain; Invitrogen, Carlsbad, CA, USA) and Lucifer yellow (LY; Sigma-Aldrich). LY with concentrations of 100 μg/ml and fluorescent *E. coli* with multiplicity of infection of 20:1 were added to the apical side of cells and incubated for 2 and 6 h at 37 °C, respectively. Monolayer permeability was assessed by measuring the fluorescence in the basal medium compartment of LY spectrophotometrically using SpectraMax M5 spectrofluorometer (Molecular Devices, Sunnyvale, CA, USA) at excitation and emission spectra of 427 nm and 536 nm, and data were reported as relative fluorescent units. The number of fluorescent *E. coli* per cross-section field was determined by inverted microscopy (Olympus, Japan).

### Immunofluorescence analysis

The paraffin-embedded intestine sections (6 μm) were dewaxed and rehydrated. After overnight antigen retrieval, the sections were incubated with block buffer (5% bovine serum albumin in PBS) for 1 h at room temperature. The sections were stained with anti-claudin-1 (1:100) or anti-occludin (1:100) antibody at 4 °C overnight, followed by Alexa-Fluor-488-conjugated anti-rabbit IgG (1:2000) antibody. To identify nuclei, tissues were counterstained with the fluorescent dye 4′,6-diamidino-2-phenylindole for 5 min. In all cases, antibody-negative controls were evaluated to ensure that the results were not a consequence of cross reactivity or nonspecific binding of the secondary antibodies. All images were measured using a laser scanning confocal microscope (Zeiss LSM 510 META; Carl Zeiss, Oberkochen, Germany).

### Western blotting

The frozen intestine (~ 100 mg) was pulverized in liquid nitrogen and suspended in 400 ml cell lysis buffer. The homogenates were sonicated for 15 s. The tissue lysate supernatants were extracted after centrifugation for 15 min at 13,500×*g* at 4 °C. Cells were harvested and lysed in the lysis buffer. The lysates were centrifuged at 12,000×*g*, for 5 min at 4 °C. Protein concentration was measured using the Bradford protein assay (Bio-Rad, Hercules, CA, USA). Equal amounts of total protein were loaded and electrophoresed through SDS-PAGE and transferred to polyvinylidene difluoride membranes (Millipore) for 2 h at 4 °C. After blocking in 5% nonfat milk for 1 h at room temperature, the membranes were treated with anti-claudin-1 (1:1000 dilution), anti-occludin (1:1000 dilution), anti-claudin-2 (1:1000 dilution), or mouse anti-β-actin (1:1000 dilution) antibody at 4 °C overnight. Secondary antibodies were HRP-conjugated goat anti-rabbit IgG (Cell Signaling Technology) or goat anti-mouse IgG (Cell Signaling Technology). Densitometric analysis was performed using an image analysis program (FluorChem8900; Alpha Innotech Corp., San Leandro, CA, USA).

### Quantification of gene expression using real-time PCR

Total RNA from tissue samples or cultured cells was extracted using the Transcriptor First Strand cDNA Synthesis Kit (Roche). Real-time PCR for mRNA expression of TJ proteins, including claudin-1, claudin-2, and occludin, was performed using the FastStart Universal SYBR Green Master (ROX) Kit (Roche) with an ABI Prism 7900 H T Sequence Detection System (Life Technology). The primer sequences for real-time PCR analysis are shown in Additional file 1: Table S1. All reactions were conducted in triplicate. β-Actin was used as an internal control. Fold changes in gene expression were calculated using the 2^−ΔΔCt^ method.

## Results

### In vivo study: effects of LK diet on intestinal barrier in mice

#### Ingestion of LK diet results in serum potassium decrease

**Fig. 1:**
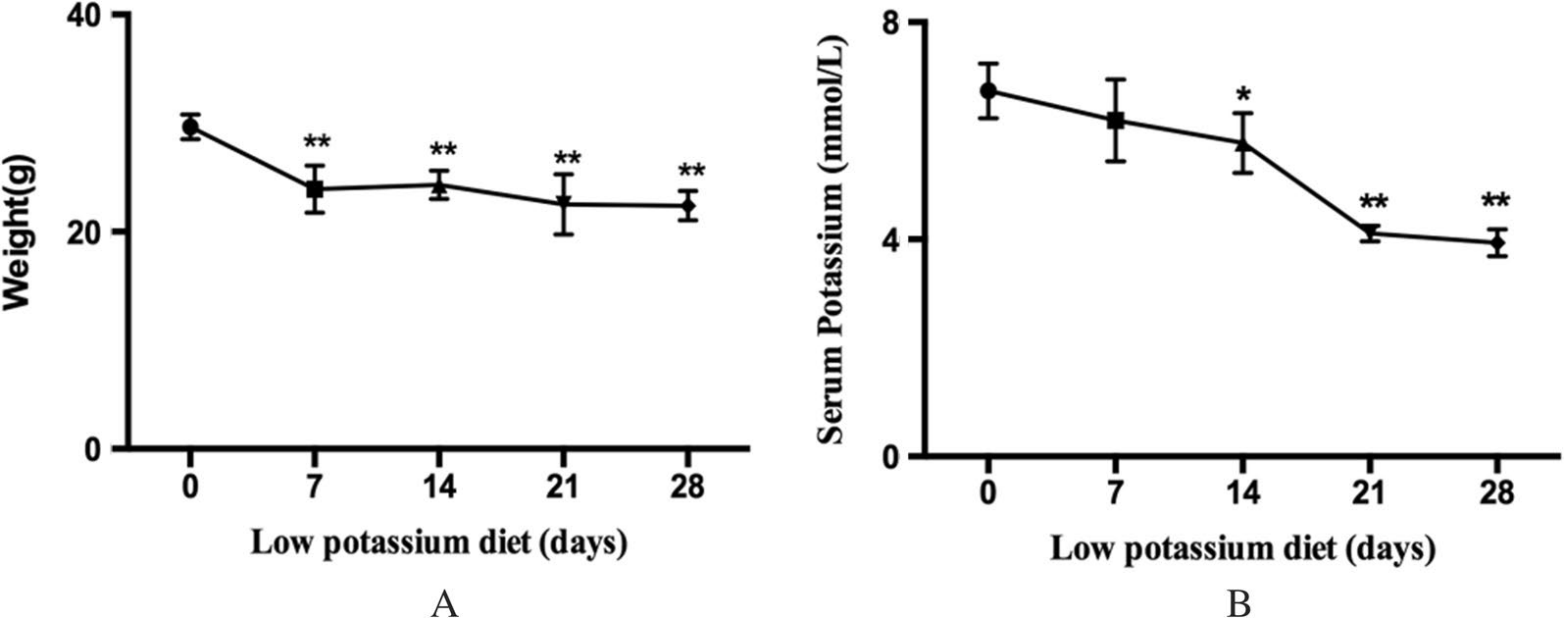
Change of weight (**A**) and serum potassium (**B**) between NK and LK diet groups. Results are presented as mean ± SD, n = 6 mice per group (*p < 0.05, **p < 0.01)

Body weight was lower in LK compared with NK diet mice after 1 week and remained significantly lower at 2 and 3 weeks (Fig. 1A). Serum potassium declined progressively over time in LK diet mice, reaching 50% decreased serum potassium compared with NK fed controls at week 3 (Fig. 1B). We used an LK diet for 28 days as our disease model group in subsequent experiments.

#### Effect of LK diet on intestinal barrier in mice

Intestinal barrier function was evaluated ex vivo by detecting the levels of endotoxin, d-lactic acid and FITC–dextran in blood. The levels of serum endotoxin (1.83 ± 0.34 vs. 0.01 ± 0.01 ng/ml, *p* < 0.01), d-lactate (193.3 ± 8.9 vs. 98.3 ± 14.3 µg/ml, *p* < 0.01) and plasma FITC–dextran (2376 ± 2489 vs. 78 ± 33 µg/ml, *p* < 0.05) in the LK diet group were significantly higher than those in the NK diet group. LK diet significantly increased paracellular permeability. In the NK diet group, the bacteria were only cultured in the MLN, and the positive rate was 16.7%. In the LK diet group, the bacteria cultured positive rate was higher, with the highest positive rate in MLN, accounting for 66.7%, followed by liver (33.3%) and spleen (16.7%).

#### Intestinal histopathological evaluation

**Fig. 2:**
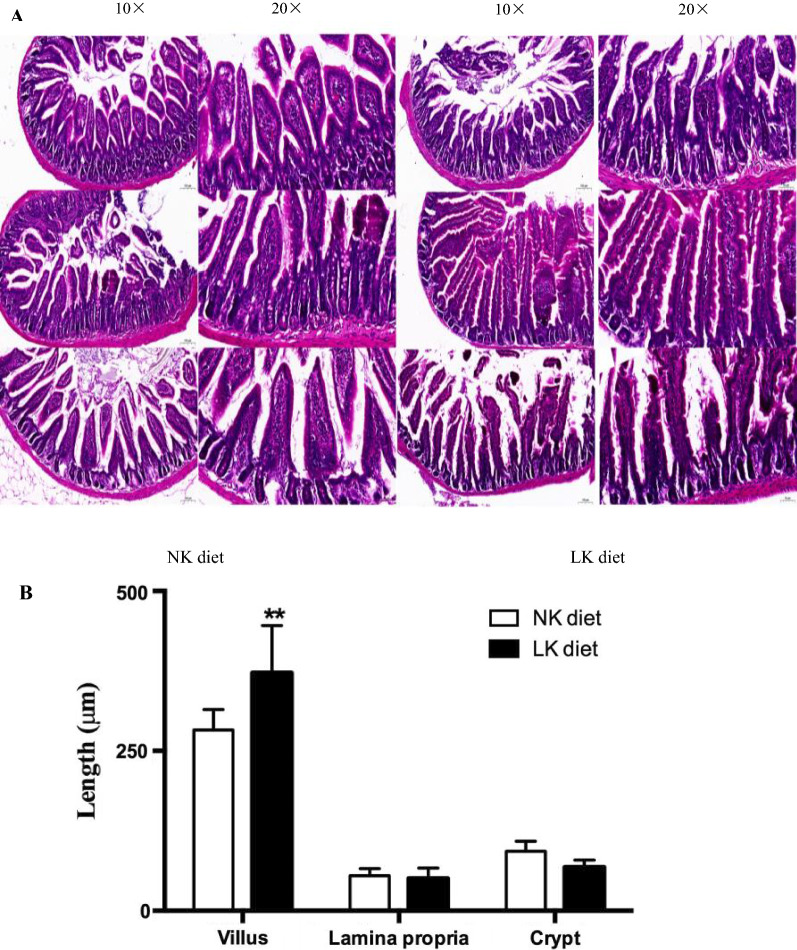
Histological analysis of intestinal in NK and LK groups. **A** HE staining of colon morphology: villus lamina propria, crypt depth and intestinal wall. **B** Histogram Bar of colon morphology

Histological measurement of colonic morphology revealed significant effects of LK diet on the intestine (Fig. 2A). Villus lamina propria thickness, crypt depth and intestinal wall were decreased in mice fed an LK diet compared with NK fed controls (Fig. 2B).

#### Effect of LK diet on intestinal movement and bacterial changes

**Fig. 3:**
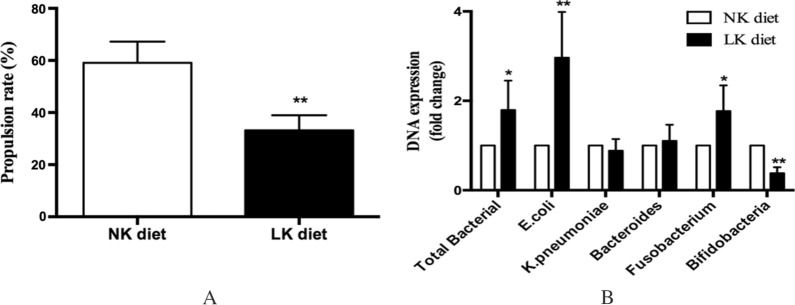
Effect of LK diet on intestinal movement and microbacterial changes. **A** Intestinal propulsive rate of activated carbon. **B** Microbacterial Change of fecal. Results are presented as mean ± SD, n = 6 mice per group. (*p < 0.05, **p < 0.01)

Compared with the NK diet group, the small intestinal propulsive rate of activated carbon was significantly lower in the LK diet group (33.2 ± 5.8 vs. 59.1 ± 8.2%, *p* < 0.01, Fig. 3A). Analysis of fecal bacteria by Q-PCR showed that total bacteria, *E. coli* and *Fusobacterium* in the LK diet group were significantly higher than those in the NK diet group with fold changes of 180 ± 65%, 296 ± 103% and 177 ± 57%, respectively. The number of Bifidobacteria was significantly lower, with a fold change of 38 ± 13% (*p* < 0.05) (Fig. 3B).

#### Changes in expression and distribution of TJ proteins in the intestine in LK diet mice

**Fig. 4:**
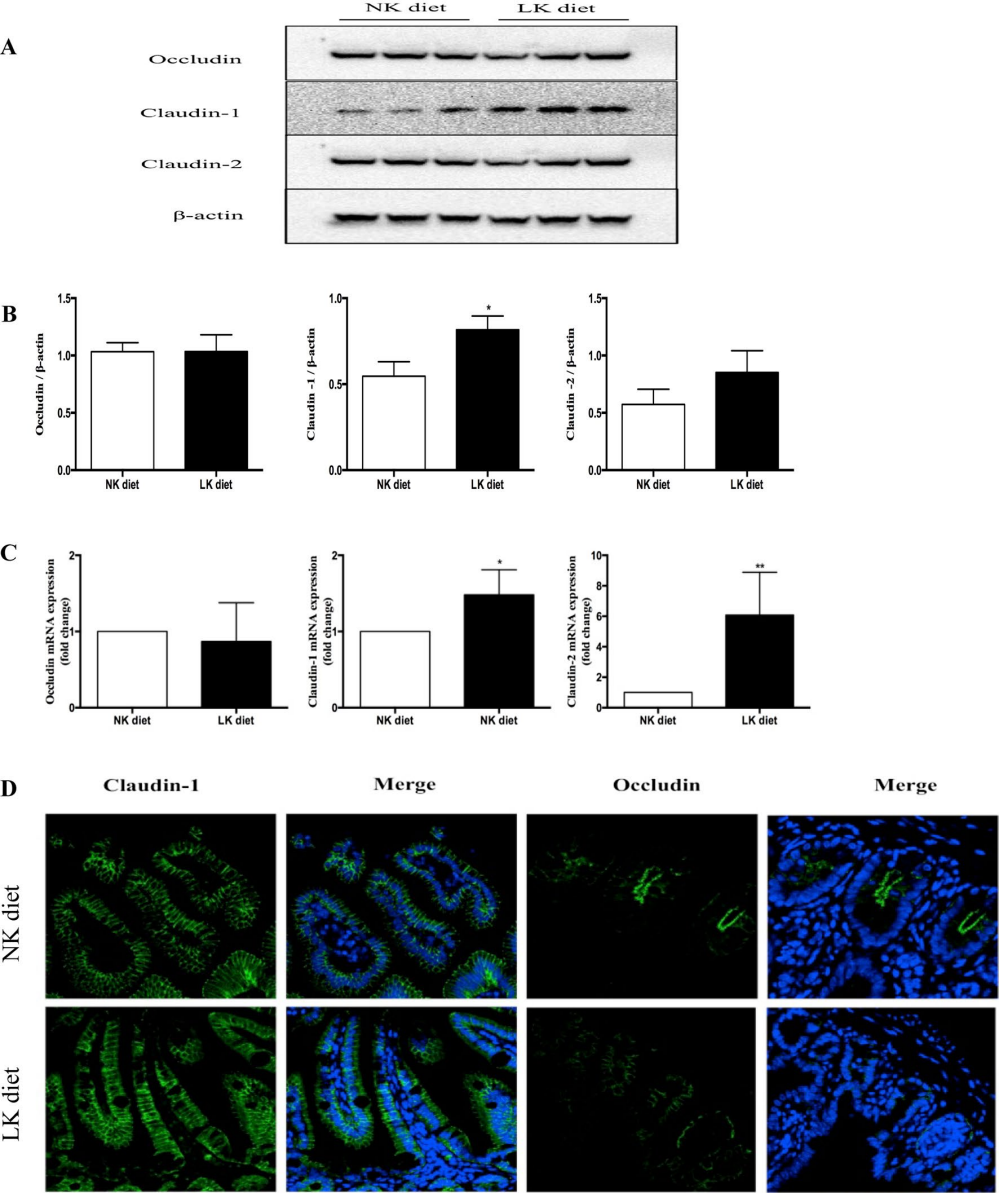
LK diet change the expression of TJ proteins. **A** Representative Western-blotting. **B** Densitometric analysis of Claudin-1, Claudin-2 and Occludin proteins expression were normalized to β-actin content. **C** The mRNA level of Claudin-1, Claudin-2 and Occludin. **D** Representative confocal microscopic images showed the localization of Claudin-1 and Occludin by indirect immunofluorescence staining in the control group and LK group. Blue corresponds to nuclear staining and green corresponds to Claudin-1 or Occludin staining. Original magnification ×400. Data are reported as means ± SD, n = 6 mice per group. *p < 0.05, **p < 0.01 vs. the control group (for interpretation of the references to color in this figure legend, the reader is referred to the web version of this article)

We examined intestinal TJ protein expression at mRNA and protein levels. Additionally, immunofluorescence was used to assess claudin and occludin localization. Real-time PCR and western blotting showed that expression of claudin-1 increased in the LK diet group, whereas occludin showed no difference in expression (Fig. 4A–C). Strong expression of claudin-1 was detected in colonic epithelial cells by immunofluorescence staining in the NK diet group. In the LK diet group, claudin-1 showed stronger expression. Occludin was predominantly expressed on the top of colonic crypts and the base of colonic epithelial cells in the NK diet group, but showed decreased staining in the LK diet group (Fig. 4D).

### In vitro study: effects of LK on Caco-2 cell permeability

#### Effect of different potassium concentrations on Caco-2 cell permeability

**Fig. 5:**
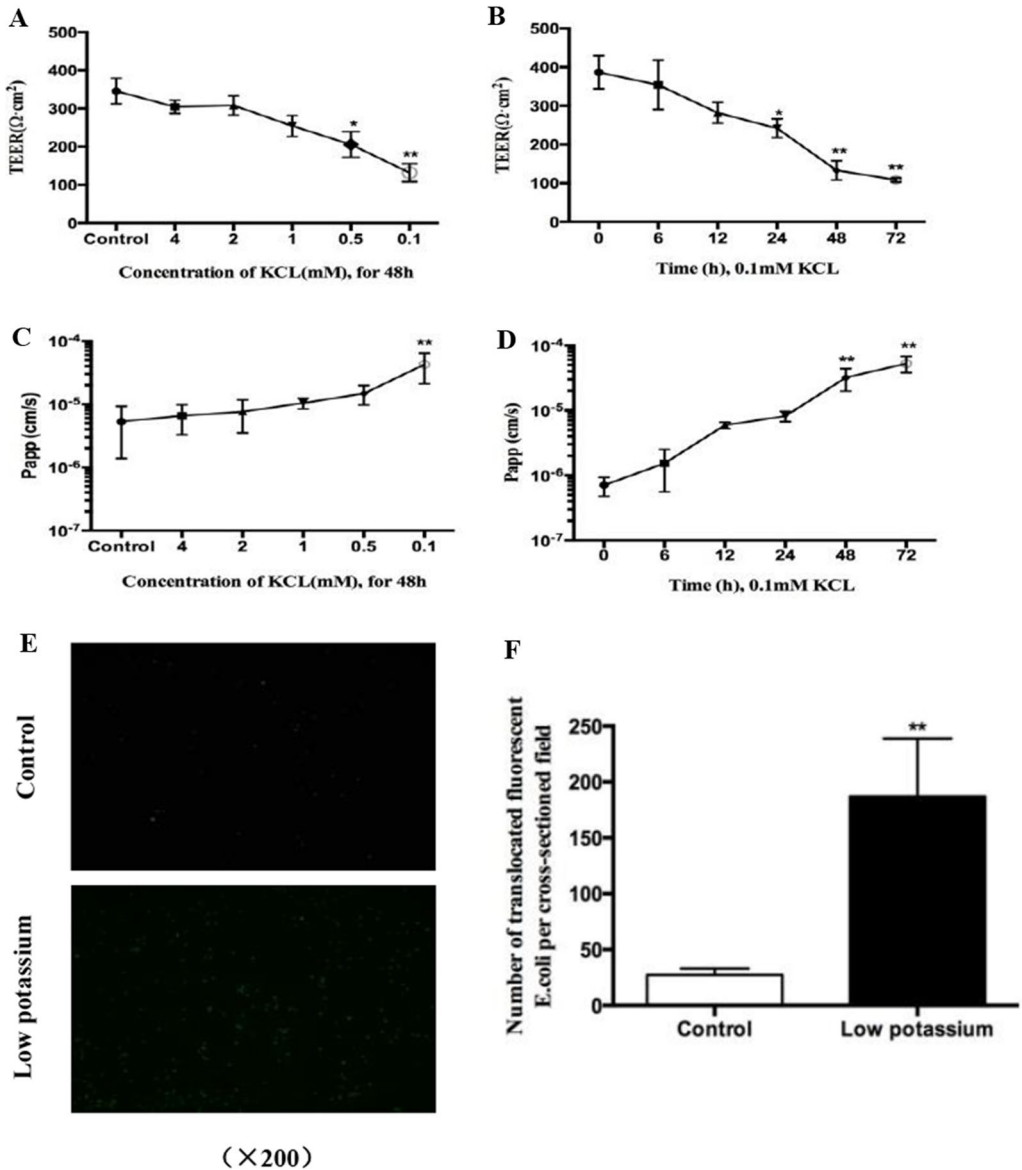
Low potassium increased permeability in Caco-2 cell. **A**, **B** Caco-2 cell monolayers were treated with low potassium medium for indicated concentration and period, TEER were measure. **C**, **D** After treated with low potassium medium for indicated concentration and period, Lucifer yellow (LY) with concentrations of 100 µg/ml were added to the apical side of cells and incubated for 2 h, permeation of LY in the basal side of cell were measured. **E** Caco-2 cell monolayers were pretreated with low potassium (0.1 mM) for 48 h, followed by incubated with flourescent *E. coli* (K-12 strain, green) for 6 h, the number flourescent *E. coli* (green) per cross-section field in determined by inverted microscope. **F** Graphs display quantitation of the number of flourescent *E. coli* per cross-sectioned in the basal layer. Value are means ± SD, n = 3. *p < 0.05, **p < 0.01 vs. the control group

TER, a measure of the ion permeability of TJs, was drastically decreased by LK. The TER of untreated control cells was 387 ± 43 Ω cm^2^, but dropped to 242 ± 24 Ω cm^2^ within 24 h in the LK group and to 133 ± 25 Ω cm^2^ after 48 h. In LK-treated cells, TER decreased in a dose-dependent manner and reached significance at 0.5 mM and 0.1 mM (*p* < 0.05) (Fig. 5A, B), indicating that LK led to increased ion permeability of TJs in Caco-2 cells. TJ permeability to nonionic molecules in Na–K-ATPase-inhibited cells was determined by tracer studies using LY and fluorescent *E. coli*. LK increased TJ permeability for both LY (Fig. 5C, D) and fluorescent *E. coli* (1.9 × 10^5^ in control vs. 2.7 × 10^4^/per cross-sectioned field in low potassium, *p* < 0.01, Fig. 5E, F). These results demonstrated that LK increased the permeability of ionic and nonionic molecules through the paracellular space in Caco-2 cells.

#### Effect of LK on Caco-2 cell expression of TJ proteins

**Fig. 6:**
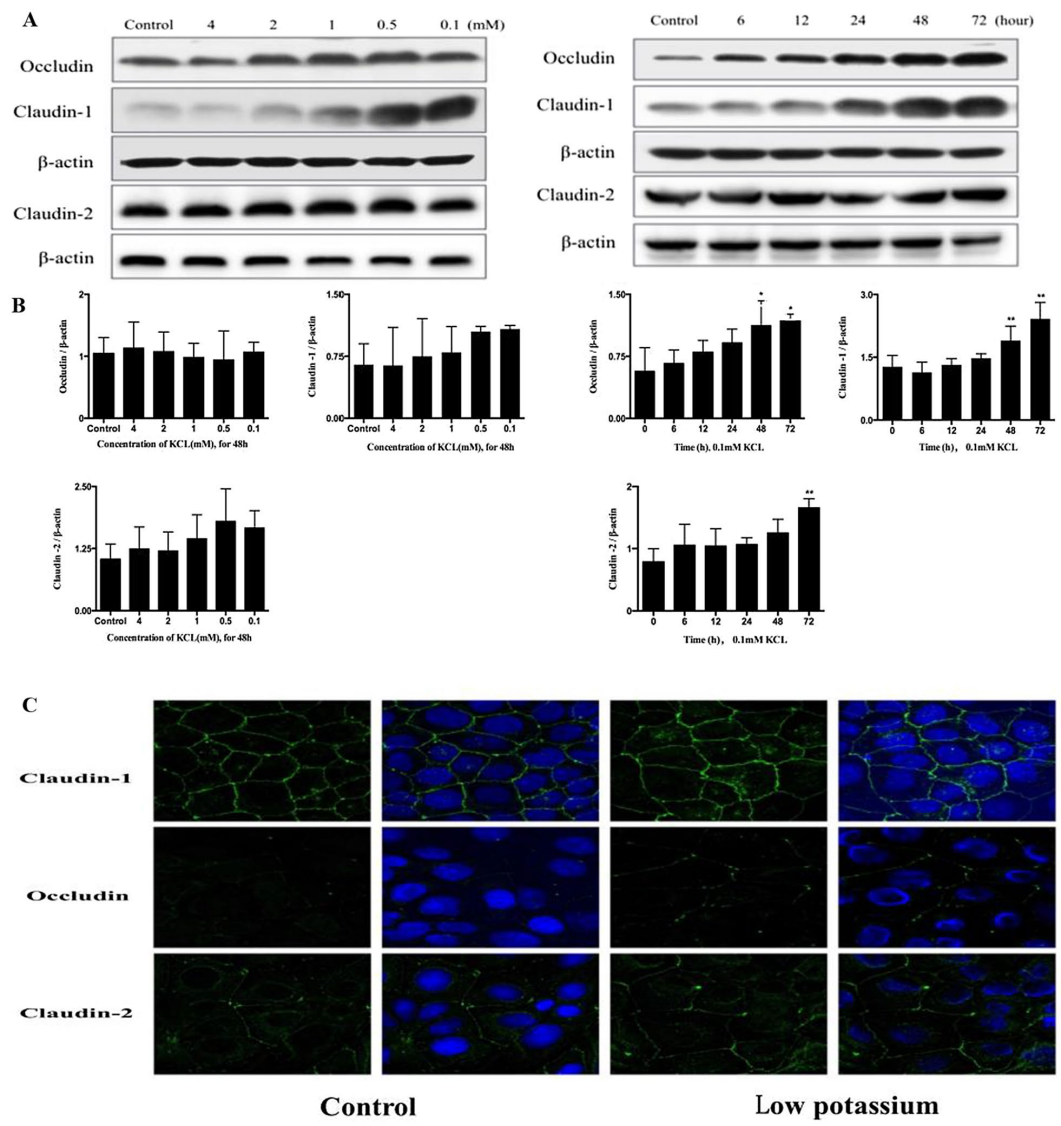
Low potassium disrupt the tight junctions in Caco-2 cell. **A** Western blot analysis of Claudin-1, Claudin-2 and Occludin in Caco-2 cells treated with low potassium at various concentrations for 48 h or 0.1 mM potassium for the indicated time periods. β-Actin was used as a loading control. **B** Densitometric anaysis of the blots showing the ratios of Claudin-1, Claudin-2 and Occludin to β-actin. **C** Immunofluorescence staining of Claudin-1, Claudin-2 and Occludin in Caco-2 cell (original magnification ×800, all sections). Values are means ± SD, n = 3. *p < 0.05, **p < 0.01 vs. the control group

We tested whether the observed permeability changes were associated with altered TJ organization. Following treatment of Caco-2 cells with potassium at concentrations of 0.1, 0.5, 1.0, 2.0, 4.0 and 5.33 mM for 48 h, western blotting demonstrated the expression of claudin-1 and claudin-2 has a trend of dose-dependent increment, while the expression of occludin did not change significantly. Following treatment with 0.1 mM potassium for 0 (control), 6, 12, 24, 48 and 72 h, expression of claudin-1, claudin-2 and occludin increased in a time-dependent manner (Fig. 6A, B). In accordance with western blotting data, LK enhanced immunofluorescence staining of claudin-1 and claudin-2 (Fig. 6C).

## Discussion

The present study provides evidence that LK diet can increase intestinal permeability and then lead to BT, which are associated with intestinal epithelial barrier injury, intestinal dysbacteriosis and abnormal intestinal peristalsis.

The intestinal tract contains large numbers of bacteria, and bacteria in the body and intestines coexist peacefully through the synergy of various intestinal defense mechanisms to form a mutually beneficial symbiotic relationship. The intestinal barrier can prevent bacteria and their toxins from translocation out of the intestinal cavity. Ischemia, hypoxia, acidosis and inflammatory mediators collectively induce necrosis of epithelial cells, impair the intestinal mucosal barrier, increase intestinal permeability, and cause bacterial and endotoxin translocation [4]. Previous clinical studies have observed that PD patients with hypokalemia have a high prevalence of peritonitis [13]. The mechanisms for the increased risk of peritonitis caused by LK is speculated to be associated with malnutrition, impaired immune function and decreased intestinal motility [22]. Our results confirm that animals fed an LK diet have increased intestinal permeability and occurrence of BT.

The epithelium is the main defensive barrier of the intestine, which inhibits the free passage of pathogens to protect the intestine [23]. Disturbed intestinal integrity and permeability may lead to the leakage of bacteria or their metabolites into the circulation, or even BT. Impaired intestinal epithelial barrier function in pathological conditions has been associated with reduced expression and changes in distribution of TJ proteins. At present, there is no consistent conclusion about the relationship between the expression of TJ proteins and intestinal mucosal permeability. Many studies have confirmed that the abnormal expression of claudin family proteins is associated with the decline of intestinal barrier function. In most studies, increased intestinal permeability is often accompanied by decreased expression of claudin-1 or occludin, and increased expression of pore-forming protein claudin-2 [24–26]. However, Xu et al. found that expression of claudin-1 increased significantly during the inflammatory phase of inflammatory bowel disease, with increased permeability, while in the chronic recovery period, expression of claudin-1 decreased gradually [27]. Although claudin-2 belongs to the claudin family, it has a different role in the epithelial barrier from claudin-1, which is a pore-forming protein. Increased expression of claudin-2 is considered to be one of the reasons for increased intestinal permeability. In decompensated cirrhosis, expression of claudin-2 is significantly increased, suggesting that this is associated with increased permeability [28]. In the present study, the mechanism of action of LK on TJ was still unknown. The possible factors for this complex regulatory mechanism include: different basic activities of signaling pathways; different dynamics of claudin protein response; different duration of action; and different turnover of connexins in various steps, such as transcription level and protein stability/degradation [29]. Inhibition of Na–K-ATPase activity by K^+^ depletion increased permeability of ionic and nonionic molecules in HPAF-II cells by regulating occludin phosphorylation, while expression of occludin and claudin-4 did not differ significantly [17]. Further studies are needed to establish the particular signaling pathway of LK on TJs.

Intestinal dysbiosis and bacterial adherence are other factors involved in the pathogenesis of BT. Dysbiosis is the shift in the microbiota composition in which there is a decrease in the number of beneficial bacteria and an increase in potentially pathogenic bacteria [30]. Stable microecosystems improve intestinal permeability by upregulating expression of TJ proteins, and promote the development of mucosal immunity to jointly resist pathogen invasion [31, 32]. However, when the intestinal microecological balance is destroyed, opportunistic pathogenic bacteria become the dominant flora, intestinal microflora biodiversity decreases, and bacterial overgrowth leads to increased intestinal epithelial permeability [33]. Breath hydrogen test is a commonly used method to detect intestinal bacterial overgrowth in the clinic. The breath hydrogen test in PD patients found that the proportion of abnormal results in hypokalemia patients was higher than that in continuous ambulatory PD patients with normal serum potassium, suggesting that intestinal bacterial overgrowth exists in hypokalemia patients [34]. In the present study, intestinal dysbacteriosis in mice fed an LK diet also increased intestinal permeability. Normal intestinal peristalsis is also an essential factor for maintaining the balance of the intestinal barrier. Normal intestinal peristalsis prevents excessive proliferation and adhesion of pathogenic intestinal bacteria. Inhibition of intestinal motility can lead to intestinal barrier dysfunction [35] and increase the development of BT. It has been shown that the application of gastrointestinal motility drugs is helpful for prevention and treatment of BT. Possible mechanisms may be related to improved permeability by accelerating intestinal motility, thus reducing the production of bacteria and endotoxin. The findings of the present study indicated that damage of the intestinal barrier in the LK environment was involved in destruction of intestinal mucosal barrier function.

## Conclusions

In conclusion, the increase of intestinal permeability mediated by LK may cause BT, which is suspected to result from impaired intestinal epithelial barrier function. These results suggest appropriate dietary potassium intake on an individual basis to maintain serum potassium level and regulation of intestinal flora homeostasis may improve intestinal barrier function. Further experiments should be carried out to determine the mechanisms that LK mediates the expression of TJ proteins and intestinal dysbacteriosis.

## Supplementary Information

**Additional file 1: Table S1.** Primer sequences for quantitative real-time PCR.

## Data Availability

Not applicable.

## Abbreviations

CKD: Chronic kidney disease
NK: Normal potassium
LK: Low potassium
MLN: Mesenteric lymph nodes
BT: Bacterial translocation
TJs: Tight junctions
PD: Peritoneal dialysis
PCR: Polymerase chain reaction
PBS: Phosphate-buffered saline
HE: Hematoxylin–eosin
TER: Transepithelial electrical resistance
