# Supplementary material for: Low potassium disrupt intestinal barrier and result in bacterial translocation
Source: J Transl Med. 2022 Jul 6;20:309. doi: 10.1186/s12967-022-03499-0 (PMC9258207; doi:10.1186/s12967-022-03499-0)
Supplement: Supplementary file 1 — Additional file 1: Table S1. Primer sequences for quantitative real-time PCR. [file 12967_2022_3499_MOESM1_ESM.docx]

**Table S1** Primer sequences for quantitative real-time PCR.

| Gene | Primer sequences |
| --- | --- |
| General sequence of Bacteria | **Forward:** CTCCTACGGGAGGCAGCAGT  **Reverse:** ATTACCGCGGCTGCTGGCAC |
| Escherichia coli | **Forward:** ACGGTAACAGGAAGCAGCTT  **Reverse:** CCCTCTTTGGTCTTGCGA |
| Kleiber pneumonia | **Forward:** GATGAAACGACCTGATTGCATTC  **Reverse:** CCGGGCTGTCGGGATAAG |
| Bacteroides | **Forward:** GCAGCATATTTGTAGCAATACAGAT  **Reverse:** CTATACCATCGGGTATTAATCTTTC |
| Fusobacterium | **Forward:** TAATGACGGTACTTTGGAGGA  **Reverse:** CGTAGCCCTTTCTACCTGC |
| Bifidobacteria | **Forward:** GAGCAAGCCTTCGGGTGAGT  **Reverse:** GGCCCCACATCCAGCGTC |
| Reference gene for Bacteria | **Forward:** AGCGAAAGACAGGTGAGAATCC  **Reverse:** CCTATCGGCCTCGGCTTAG |
| Claudin-1 | **Forward:** TGCCCCAGTGGAAGATTTACT  **Reverse:** CTTTGCGAAACGCAGGACAT |
| Occludin | **Forward:** TGAAAGTCCACCTCCTTACAGA  **Reverse:** CCGGATAAAAAGAGTACGCTGG |
| Claudin-2 | **Forward:** AGGTCGGTGTGAACGGATTTG  **Reverse:** TGTAGACCATGTAGTTGAGGTCA |
| β-actin | **Forward:** GGCTGTATTCCCCTCCATCG  **Reverse:** CCAGTTGGTAATGCCATGT |
